# MITOPHAGY ACTIVATION EXTENDS LONGEVITY AND PREVENTS AGE-RELATED CARDIAC FUNCTIONAL DECLINE

**DOI:** 10.1093/geroni/igae098.0735

**Published:** 2024-12-31

**Authors:** Nuo Sun

**Affiliations:** Ohio State University, Columbus, Ohio, United States

## Abstract

Mitophagy is a specialized autophagic pathway that removes damaged mitochondria, providing a critical mechanism to maintain mitochondrial quality. Accumulating evidence suggests that a decline in mitophagy might contribute to the development of age-related cardiac pathologies. Therefore, pharmacological manipulation of mitophagy offers a novel therapeutic strategy to alleviate the risks of disease and mortality associated with aging. We conducted high-content image-based assays for mitophagy modulators using the pH-dependent fluorescent mitophagy reporter, mt-Keima. Additionally, we designed an algorithm to analyze a collection of over 10,000 compounds at 15 concentrations. This algorithm identified several selective mitophagy inducers, with Mito-I emerging as the most effective mitophagy activator. We demonstrated that Mito-I potently induces mitophagy in human cardiac myocytes derived from induced pluripotent stem cells (hiPSC-CM) and mouse hearts. Interestingly, our mechanistic studies revealed that Mito-I effectively upregulates BCL2-interacting protein 3 (BNIP3), which acts as a mitophagy receptor and is indispensable for Mito-I-induced mitophagy. Excitingly, we also found that Mito-I treatment extended lifespan in C. elegans. Moreover, we showed that Mito-I administration in aged (18-month-old) mice alleviates the detrimental effects of aging on cardiovascular health. These results demonstrate that the age-induced decrease in mitophagy contributes significantly to diminished cardiovascular function and identifies a novel therapeutic strategy to improve cardiovascular function with aging.

